# Supplementary material for: Mass Transfer Characteristics of Haemofiltration Modules—Experiments and Modeling
Source: Membranes (Basel). 2022 Jan 1;12(1):62. doi: 10.3390/membranes12010062 (PMC8780501; doi:10.3390/membranes12010062)
Supplement: Supplementary file 1 [file membranes-12-00062-s001.zip › membranes-1505763-supplementary.pdf]

Supplement to

Mass transfer characteristics of haemofiltration modules – Experiments and modeling

Table S1 presents the "corrected" *exact external pressures* at inlet/outlet of the Haemofiltration (HF) module, after subtracting or adding static pressure differences. Subscripts 1 to 4 designate the location of stream inlet or outlet, $P_1/P_{Blood\ in}$, $P_2/P_{Blood\ out}$, $P_3/P_{Dialysate\ in}$, $P_4/P_{Dialysate\ out}$. These data are needed for complete fluid-mechanical characterization of the HF module employed in the mass transfer experiments (manuscript refs [12, 13])

Figure S1 depicts typical recordings of blood- and dialysate-side inlet flow rates for the three cases studied, indicating the prevailing steady-state conditions.

In **Figures S2** to **S5,** the measured urea concentration C_D profiles for Cases II and III (for the blood-side and dialysate-side flow fields) are contrasted with theoretical profiles representing several levels of contributions of solute diffusion, by varying the parameter λ within three orders of magnitude (i.e. from 10^{-2} to 10^{0}).

As a test on the accuracy of experimental data, checks on the closure of urea mass balance for these experiments was performed; such an example is provided at the end of this document.

Table S1. Characteristic pressure differences for: (a) *Mode #3*, (b) *Mode #4*.

(a)

Q_{Blood} (mL/min)	$\Delta P = P_1 - P_4$ $[P'_1 - P'_4 + 0.06]$ (psi)	$\Delta P = P_1 - P_2$ $[P'_1 - P'_2 + 0.54]$ (psi)	$\Delta P = P_2 - P_3$ $[P'_2 - P'_3 - 0.07]$ (psi)
200	0.84±0.05	0.48±0.03	0.31±0.04
250	1.29±0.01	0.63±0.05	0.40±0.03
300	1.64±0.01	0.81±0.04	0.42±0.02
350	1.93±0.02	0.96±0.04	0.52±0.01
400	2.41±0.08	1.21±0.07	0.61±0.01
450	2.88±0.01	1.47±0.04	0.67±0.01
500	3.38±0.06	1.75±0.07	0.74±0.01

(b)

$Q_{Dialysate}$ (mL/min)	$\Delta P = P_3 - P_2$ $[P'_3 - P'_2 - 0.07]$ (psi)	$\Delta P = P_3 - P_4$ $[P'_3 - P'_4 - 0.36]$ (psi)	$\Delta P = P_4 - P_1$ $[P'_4 - P'_1 - 0.06]$ (psi)
200	0.64±0.01	0.15±0.03	0.28±0.01
300	1.22±0.01	0.27±0.01	0.42±0.01
400	1.96±0.00	0.46±0.01	0.56±0.00
450	2.23±0.01	0.53±0.01	0.64±0.00
500	2.78±0.02	0.69±0.01	0.71±0.01
550	3.20±0.02	0.81±0.02	0.75±0.01
600	3.69±0.02	0.94±0.02	0.84±0.03

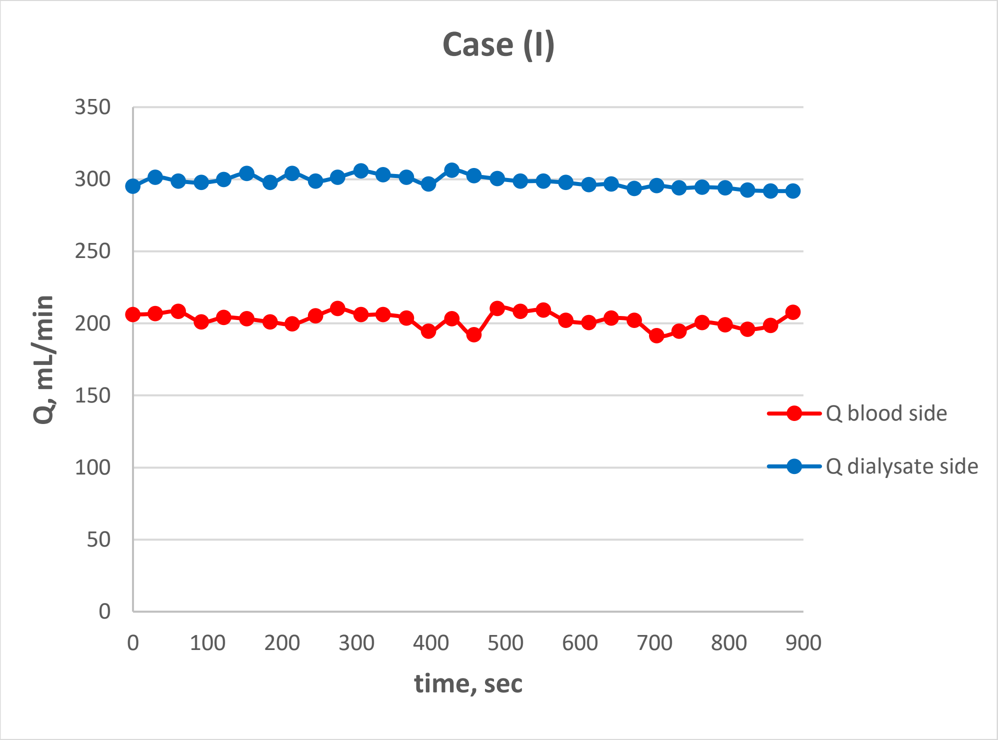

(a)

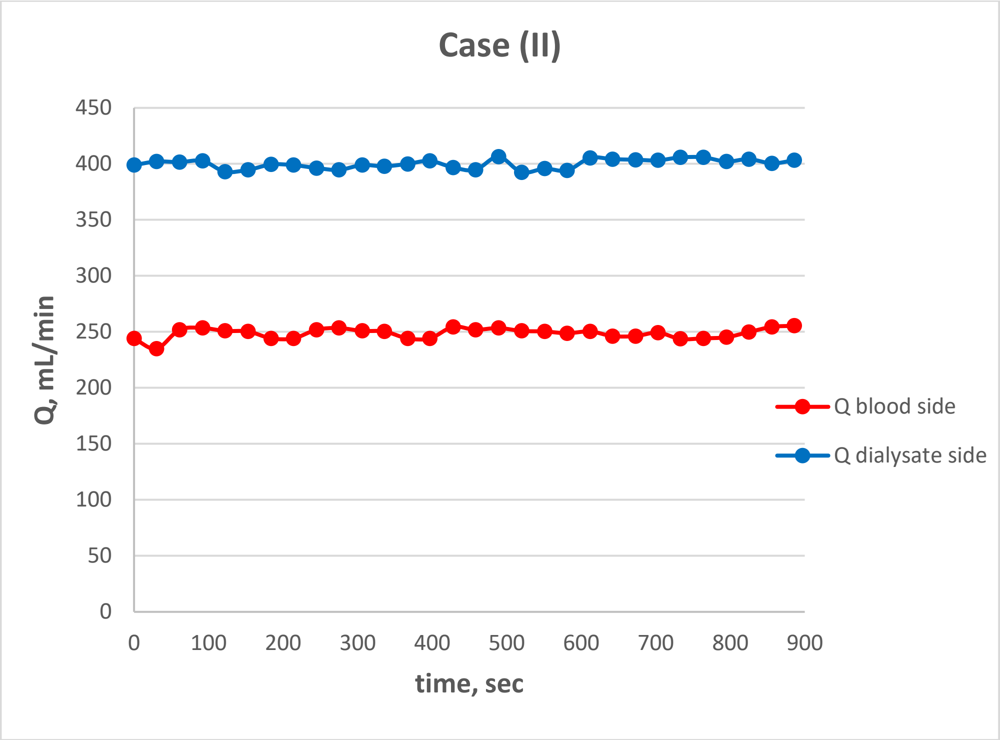

(b)

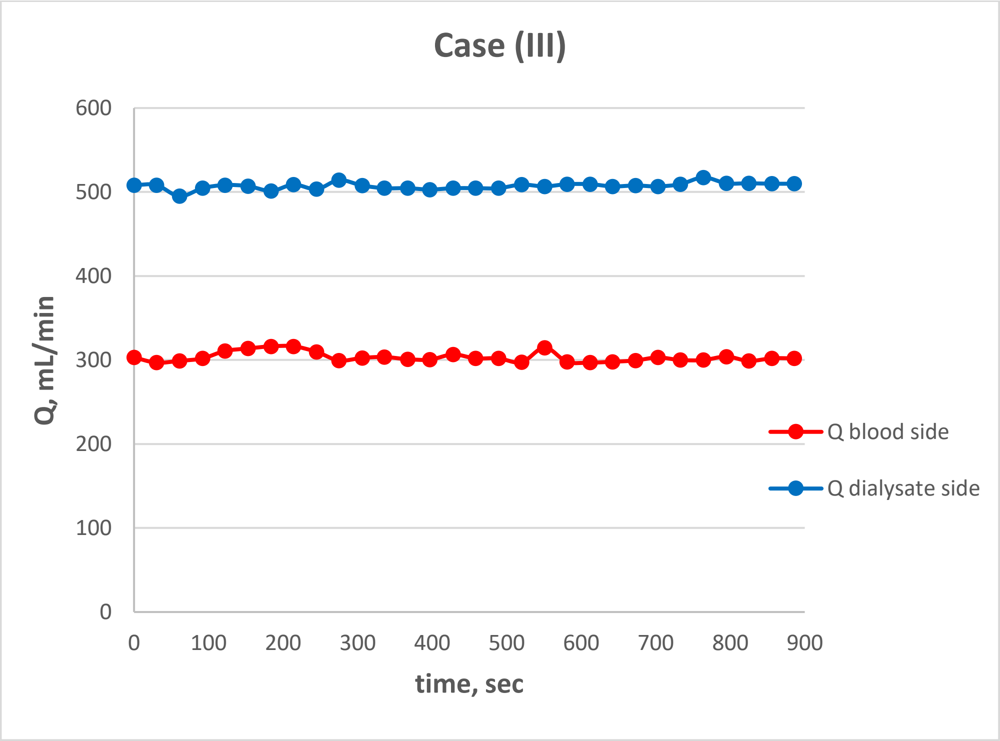

(c)

Figure S1. Typical recordings of blood- and dialysate-side inlet flow rates for the three cases studied, indicating prevailing steady-state conditions. **(a)** Case (I), $Q_{Blood}/Q_{Dialysate}$: 200/300 mL/min. **(b)** Case (II), $Q_{Blood}/Q_{Dialysate}$: 250/400 mL/min. **(c)** Case (III), $Q_{Blood}/Q_{Dialysate}$: 300/500 mL/min.

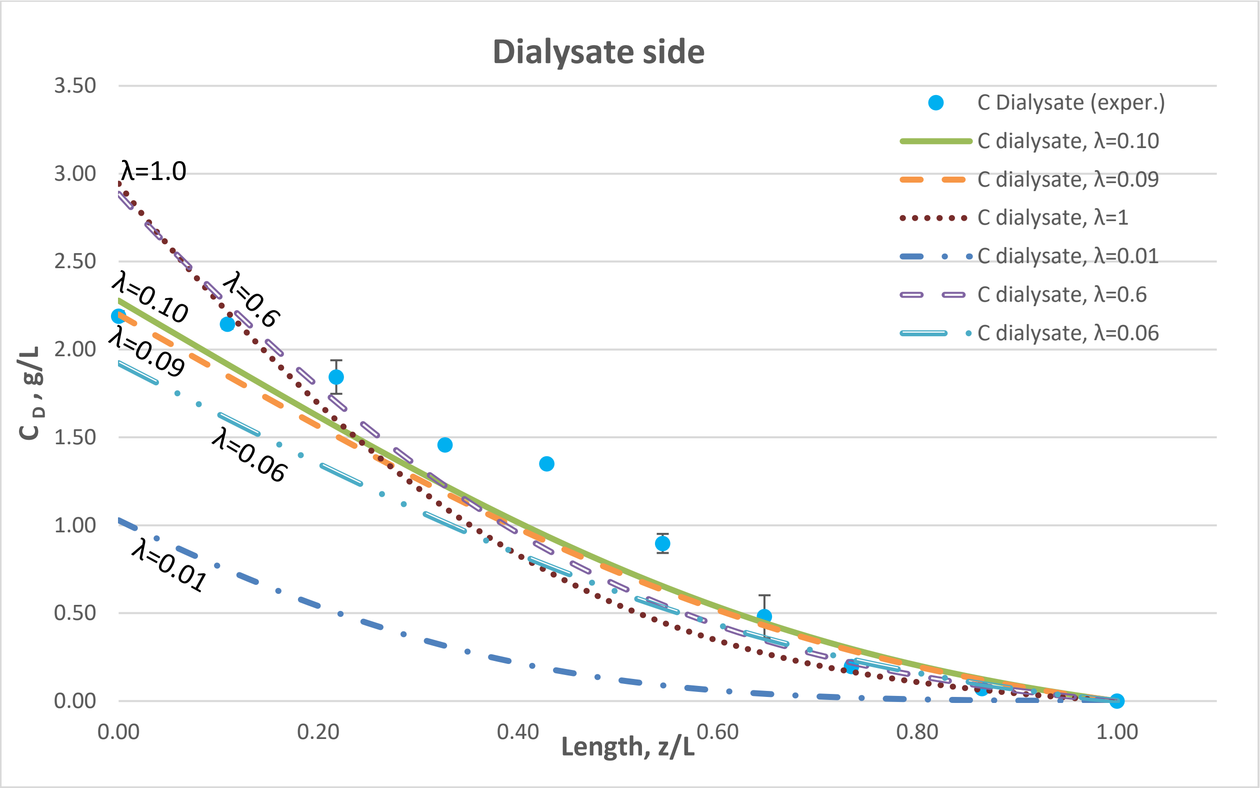

Figure S2. Dialysate-side urea concentration profile. Influence of the urea effective diffusion coefficient in the membrane (D_e) on the urea mass transfer process, under simultaneous trans-membrane liquid convection and solute diffusion. Case (II): Q_{Blood}=250 mL/min, $Q_{Dialysate}$=400 mL/min.

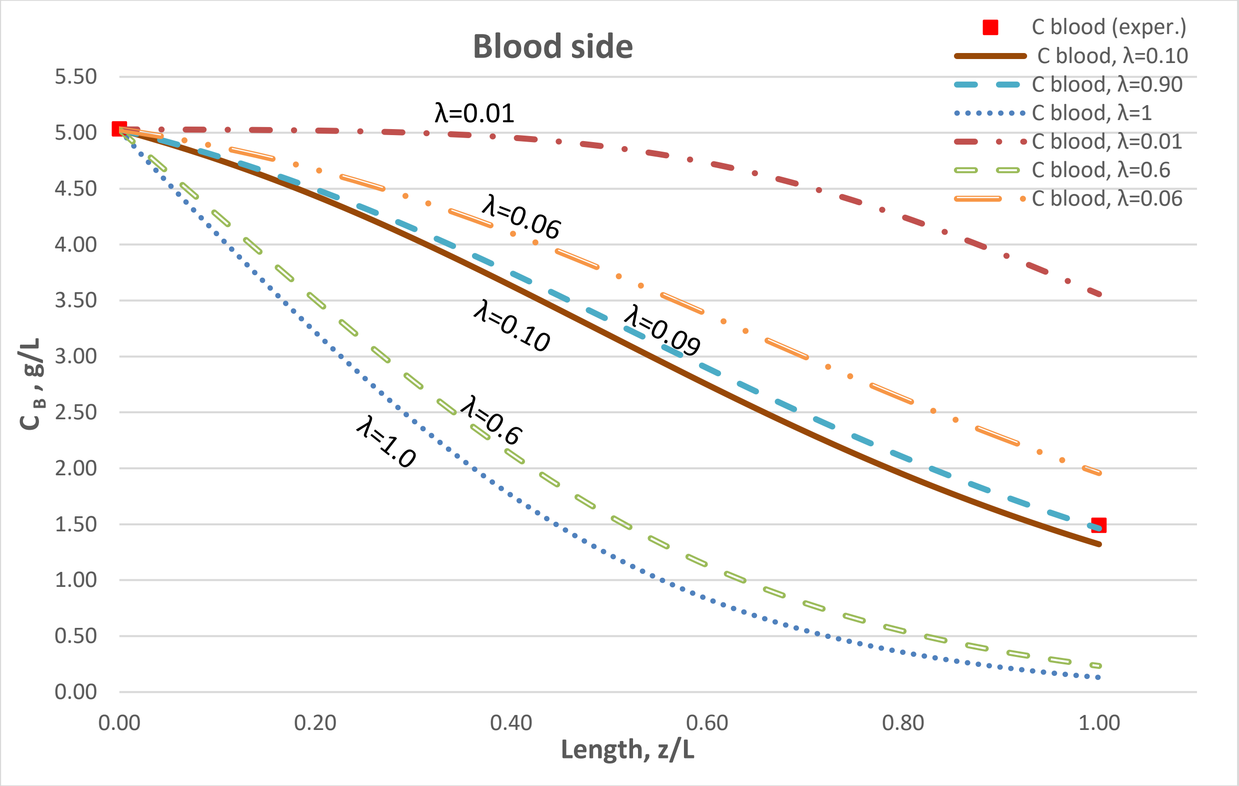

Figure S3. Blood-side urea concentration profile. Influence of the urea effective diffusion coefficient in the membrane (D_e) on the urea mass transfer process, under simultaneous trans-membrane liquid convection and solute diffusion. Case (II): Q_{Blood}=250 mL/min, $Q_{Dialysate}$=400 mL/min.

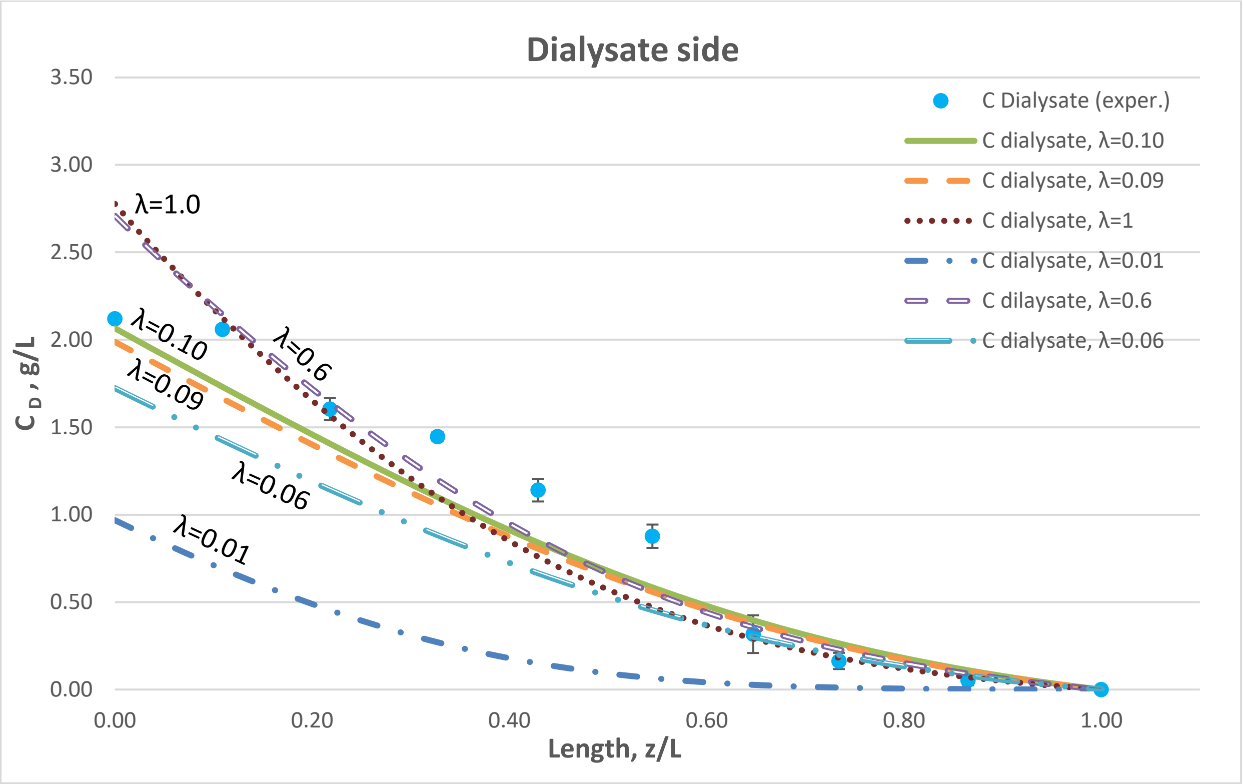

Figure S4. Dialysate-side urea concentration profile. Influence of the urea effective diffusion coefficient in the membrane (D_e) on the urea mass transfer process, under simultaneous trans-membrane liquid convection and solute diffusion. Case (III): Q_{Blood}=300 mL/min, $Q_{Dialysate}$=500 mL/min.

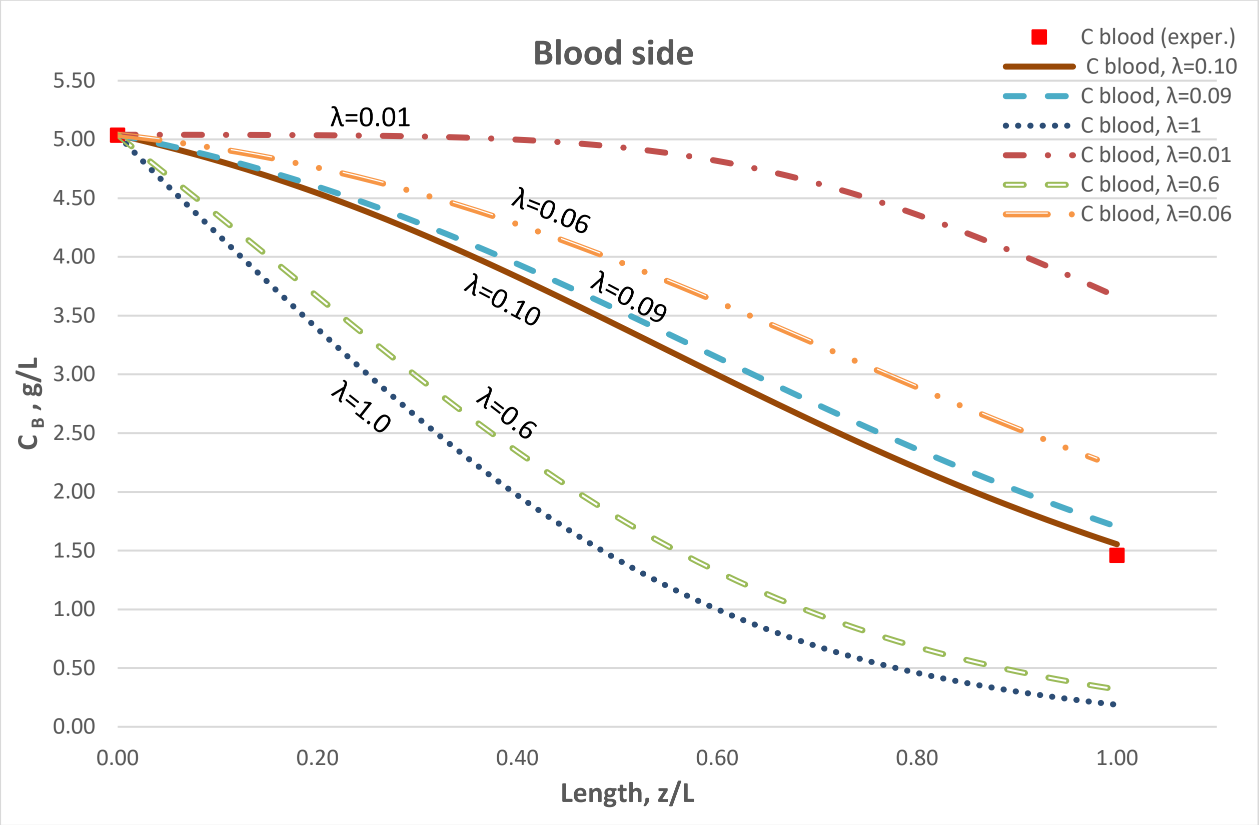

Figure S5. Blood-side urea concentration profile. Influence of the urea effective diffusion coefficient in the membrane (D_e) on the urea mass transfer process, under simultaneous trans-membrane liquid convection and solute diffusion. Case (III): Q_{Blood}=300 mL/min, $Q_{Dialysate}$=500 mL/min.

Closure of urea mass balance for the experiments

For each experiment of Cases I, II and III, closure of both volume- and mass-balance has been checked, including urea mass-balance closure corresponding to the experimental results depicted in Figures 6 - 8. For example, regarding Case I, Figure 6, by using the outlet/inlet values C_{Dout} = 1.36 and C_{Din} = 0, and the respective inlet/exit flow rates Q_D and blood-stream data (listed in Table A1) excellent closure is obtained (difference < 2 %), as shown below

$$\mathbf{C_{Bin}\,Q_{Bin} - C_{Bout}\,Q_{Bout} = C_{Dout}\,Q_{Dout} - C_{Din}\,Q_{Din}}$$

$$(5.02\bullet200) - (1.36\bullet185.93) = (2.42\bullet314.07) - 0 \quad \rightarrow \quad \mathbf{751.14 \approx 760.05}$$
